# Application of Quercetin in the Treatment of Gastrointestinal Cancers

**DOI:** 10.3389/fphar.2022.860209

**Published:** 2022-04-06

**Authors:** Seyed Mohammad Ali Mirazimi, Fatemeh Dashti, Mohammad Tobeiha, Ali Shahini, Raha Jafari, Mehrad Khoddami, Amir Hossein Sheida, Parastoo EsnaAshari, Amir Hossein Aflatoonian, Fateme Elikaii, Melika Sadat Zakeri, Michael R Hamblin, Mohammad Aghajani, Minoodokht Bavarsadkarimi, Hamed Mirzaei

**Affiliations:** ^1^ School of Medicine, Kashan University of Medical Sciences, Kashan, Iran; ^2^ Student Research Committee, Kashan University of Medical Sciences, Kashan, Iran; ^3^ Faculty of Medicine, Mashhad University of Medical Sciences, Mashhad, Iran; ^4^ Department of Medicine, Mashhad Medical Sciences Branch, Islamic Azad University, Mashhad, Iran; ^5^ Research Center for Biochemistry and Nutrition in Metabolic Diseases, Institute for Basic Sciences, Kashan University of Medical Sciences, Kashan, Iran; ^6^ Laser Research Centre, Faculty of Health Science, University of Johannesburg, Doornfontein, South Africa; ^7^ Infectious Disease Research Center, School of Nursing and Midwifery, Kashan University of Medical Sciences, Kashan, Iran; ^8^ Clinical Research Development Center, Mahdiyeh Educational Hospital, Shahid Beheshti University of Medical Sciences, Tehran, Iran

**Keywords:** gastrointestinal cancers, quercetin, therapy, natural compounds, bioactive compounds

## Abstract

Many cellular signaling pathways contribute to the regulation of cell proliferation, division, motility, and apoptosis. Deregulation of these pathways contributes to tumor cell initiation and tumor progression. Lately, significant attention has been focused on the use of natural products as a promising strategy in cancer treatment. Quercetin is a natural flavonol compound widely present in commonly consumed foods. Quercetin has shown significant inhibitory effects on tumor progression *via* various mechanisms of action. These include stimulating cell cycle arrest or/and apoptosis as well as its antioxidant properties. Herein, we summarize the therapeutic effects of quercetin in gastrointestinal cancers (pancreatic, gastric, colorectal, esophageal, hepatocellular, and oral).

## 1 Introduction

Nowadays, cancer is the major cause of death worldwide. Nearly 17 million people from 185 countries are affected by 36 different kinds of cancer, according to a report by the International Agency for Research on Cancer (IARC) and the American Cancer Society (ACS) (Miller et al., 2020; Schiller and Lowy 2021). Cancer occurs because the normal control of physiologic cell multiplication has failed, and the cells undergo mitosis many times to form a tumor mass. Moreover, cancer cells can migrate to different parts of the body to form secondary tumors or metastases. Benign tumors mostly remain limited to their site of origin and do not spread to other parts of the body, while malignant tumors, on the other hand, have a strong tendency to spread. There are three routes for metastasis: lymphatic, hematogenous, and by invasion into adjacent tissues and secondary organs (Patel 2020). The best way to improve treatment of different kinds of cancer is to diagnose the cancer at an early stage and to select the most suitable treatment option for the tumor type and stage, including surgery, radiotherapy, chemotherapy, radiochemotherapy, hormonal therapy, targeted therapy, or immunotherapy. Although there are many different kinds of treatment, the best approach is to use a combination of several types. Moreover, the physician should consider the side effects of each approach and choose an overall low-risk, high-benefit treatment (Colli et al., 2017; Schirrmacher 2019). In the past, doctors have investigated natural drugs, especially plant products, along with limited surgical interventions. There are many historical documents describing traditional medicine, such as Indian Ayurvedic practice and traditional Chinese medicine, which used plant components to treat patients. One advantage of natural products is that we have enormous resources of plant, fungal, and microbial species that can be used against cancer if they have tolerable toxicity. Natural products can be considered an opposite point of view to the present pharmaceutical-centered culture and are often considered preferable by holistic and complementary physicians. Nowadays, there are many anticancer medicines which have been obtained from plants, animals, microorganisms, and the marine environment (Chamberlin et al., 2019). McCulloch et al. and Firenzuol et al. both emphasized the need of more studies to evaluate the efficacy of natural products in cancer therapy and to compare them to standard pharmacological treatment. Moreover, it is likely that natural products will be combined with pharmaceutical drugs in the future (Firenzuoli et al., 2004; McCulloch et al., 2011). Many medicinal herbs, such as *Curcuma* species, *Cinnamomum* species, and *Artemisia* species, and their components have been used in cancer therapy (Banikazemi et al., 2021; Davoodvandi et al., 2021). Medicinal herbs can inhibit tumor cell growth, induce apoptosis, and inhibit angiogenesis (Bevara et al., 2018; Jung et al., 2018; Mayzlish-Gati et al., 2018; Zhou et al., 2019a). Flavonoids are a diverse class of phytonutrients found in almost all fruits and vegetables. They include isoflavones, anthocyanidins, flavanones, and flavonols. Although their molecular structures differ, the presence of flavonoids significantly increases the bioavailability of other compounds (Böhm 1994; Gu et al., 2004; Ding et al., 2016). Many studies have emphasized the beneficial effects of flavonoids in the daily diet and suggested that the consumption of flavonoids could be effective in reducing the risk of chronic heart and brain disorders and cancer (Kozłowska and Szostak-Wegierek 2014; Panche et al., 2016; Honari et al., 2019; Hoseini et al., 2019; Maleki Dana et al., 2021). One prominent member of the flavonoid family is quercetin (3,3′,4′,5,7-pentahydroxyflavone), which is present in different kinds of fruits and vegetables, such as buckwheat, broccoli, and onions. Nowadays, quercetin is used as a dietary supplement and could be used as an anticancer agent in the daily diet (Vargas and Burd 2010; Vafadar et al., 2020). There are many studies which demonstrate that quercetin consumption at a tolerable dosage could have beneficial biological effects, including antioxidant, anticancer, and anti-inflammatory effects (Li et al., 2016). In this article, we summarize the anticancer properties of quercetin against gastrointestinal (GI) cancers *in vivo* and *in vitro* and also assess its possible inhibitory effect on human cancers based on its cellular and molecular mechanisms.

### 2 Quercetin: Potential, Bioavailability, and Mechanisms of Action in Cancer Therapy

**FIGURE 1:**
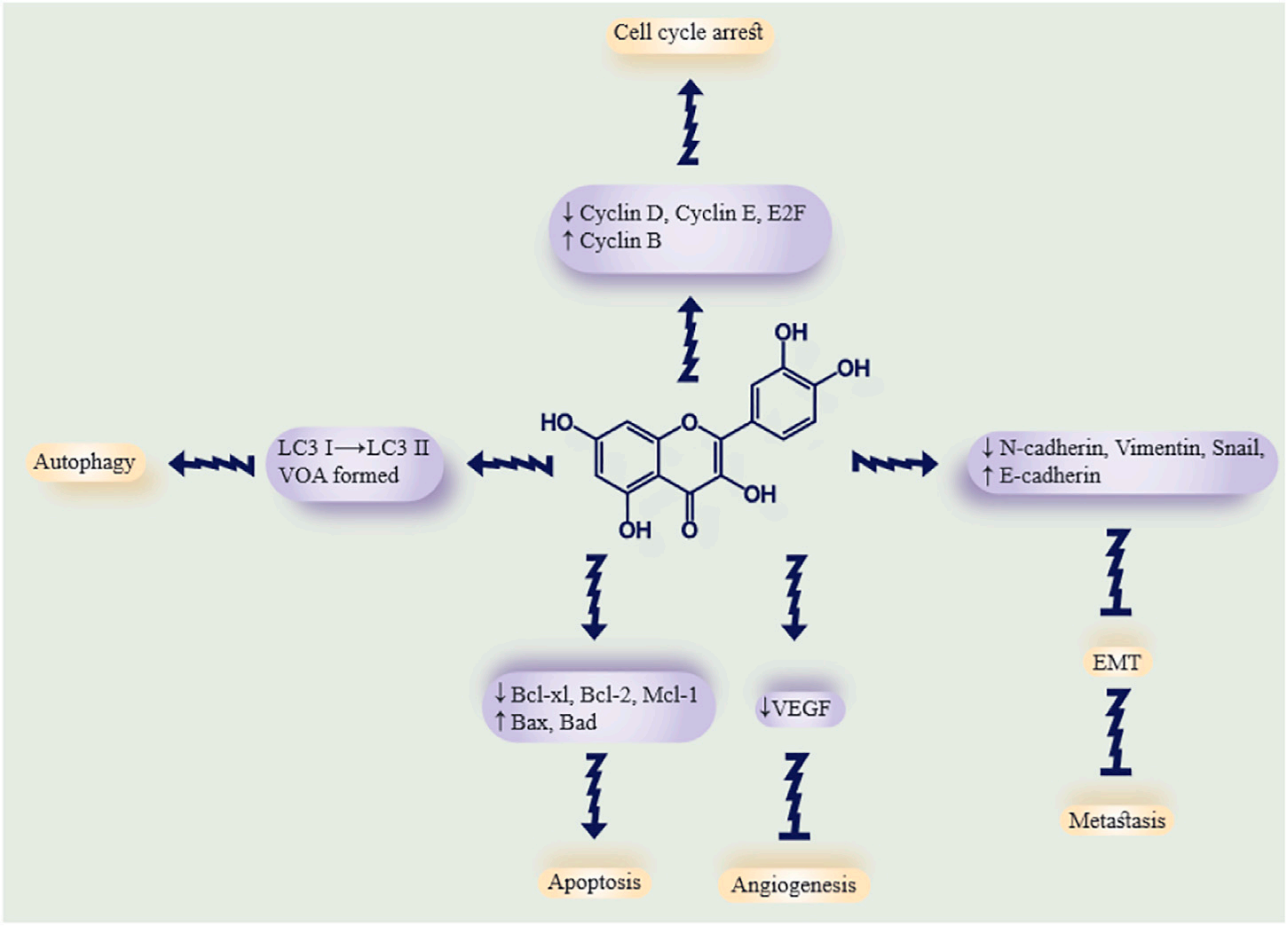
Summary of the antitumor properties of curcumin. The effects of quercetin on tumor cells and the possible molecular targets for each effect. The arrows show quercetin antitumor mechanisms, while the blunt lines show tumor-suppressive effects. Downwards or upwards arrows indicate downregulation or upregulation of molecular targets.

Quercetin is a member of the flavonoid family with a flavone nucleus, composed of a heterocyclic pyrone ring that links the two benzene rings to form the central nucleus (Russo et al., 2014). The main form of quercetin found in herbs is composed of hydrophilic glycosides (sugar conjugates), which are not easily and directly absorbed by enterocytes. The daily dosage of quercetin for human usage is 10–100 mg, which could be obtained from 500–1,000 mg of purified extract (Brito et al., 2015). Within enterocytes, quercetin undergoes a variety of enzymatic reactions, including methylation, hydrolysis, sulfonylation, and glucuronidation, by specific transferase enzymes (Brito et al., 2015). Following transportation into the intestinal lumen and then the liver, quercetin metabolites (the major quercetin-derived circulating compounds in plasma, quercetin-3-glucuronide and quercetin-3ʹ-sulfate) are formed *via* other conjugation reactions (Alrawaiq and Abdullah 2014). There are two forms of quercetin: conjugated and non-conjugated; the plasma concentration of each is 3.5–5.0 μmol/L and <0.33 μmol/L, respectively. The form of quercetin which is absorbed by enterocytes is the conjugated form (Alrawaiq and Abdullah 2014). Recently, a new study revealed that the microbes of the gut play an important role in quercetin absorption because they have enzymes which render the quercetin molecules smaller and more absorbable (Xu et al., 2017; Santangelo et al., 2019). Quercetin can be metabolized to glucuronidated, methylated, or sulfated derivatives (D'Andrea 2015). As previously stated, quercetin has several anticancer effects that have been demonstrated in numerous *in vitro* and *in vivo* studies. Its antitumor effects include its antioxidant activity, inhibition of angiogenesis, inhibition of the cell cycle and proliferation, and prevention of tumor metastasis. In addition, quercetin has many other beneficial properties that make it an effective supplement in the daily diet, including anti-inflammatory effects, antihypertensive effects, antithrombotic effects, anti-atherosclerosis properties, and anti-arrhythmia activity. Therefore quercetin has attracted attention from researchers as an adjuvant agent to take advantage of its antitumor, antioxidant, cytoprotective, and anti-proliferative properties (Lugli et al., 2009). Many studies have been conducted to evaluate the beneficial antitumor effects of quercetin against the kidney, breast, prostate, lung, ovarian, colorectal, pancreatic, and nasopharyngeal cancer (Lee et al., 2015a; Liu et al., 2017a; Baby et al., 2018; Huang et al., 2018; Li et al., 2018; Polukonova et al., 2018; Yang et al., 2019a). One of the most important features of quercetin is its pro-apoptotic effect, which is caused by increasing pro-apoptotic molecules such as P53, BAX, caspase-3, and caspase-9 or stimulating the mitochondrial apoptosis pathway or decreasing antiapoptotic proteins (Roos and Kaina, 2006; Zhang et al., 2008a; Zhang et al., 2009a; Tan et al., 2009). Quercetin could regulate and inhibit the cell cycle by activating p21 and decreasing D1/Cd4 and E/Cdk2 ratios. Quercetin can arrest the cell cycle at the G1 phase as well as inhibit microtubule polymerization which also affects the cell cycle (Gupta and Panda, 2002; Moon et al., 2003). There are various mechanisms and pathways by which quercetin can regulate the cell cycle, some of which can increase the cell cycle, such as the PI3K/AKT/PKB pathway. Moreover, quercetin can inhibit carcinogenesis and metastasis along with apoptosis induction (JÁF, 2004; Gulati et al., 2006). p53 is a key molecule in the regulation of cell death and cell survival pathways and plays a role in cancer therapy. p53 can act as an antioxidant by protecting the cellular DNA from oxidative damage and by regulating genes for endogenous antioxidants, such as catalase, Gpx1, microsomal GSH homologous transferase PIG12, Mn-SOD2, and aldehyde dehydrogenase ALDH4A1. Many research works have confirmed that quercetin can stabilize p53 levels and increase its phosphorylation (Polyak et al., 1997; Hussain et al., 2004; Yoon et al., 2004; Tanigawa et al., 2008). In malignant cells, p53 genes can be blocked or mutated, causing loss of functions (Gibellini et al., 2011). Another antioxidant function of quercetin is to quench reactive oxygen species (ROS), thereby preventing ROS-mediated DNA damage (Metodiewa et al., 1999; Awad et al., 2000). Therefore, quercetin has the potential to be used in cancer treatment because of its ability to regulate the cell cycle, antioxidant effects, p53 stabilization, and apoptosis induction. The potent anticancer activity and structure of quercetin are illustrated in Figure 1.

## 3 Quercetin and Gastrointestinal Cancer

### 3.1 Quercetin and Pancreatic Cancer

Yangyang Guo and others evaluated the effects of quercetin on pancreatic ductal adenocarcinoma (PDA) using different techniques, including a real-time cell analysis, migration, proliferation, and invasion, and used a nude mouse tumor formation assay. They evaluated tumor growth and metastasis as well as colony formation, cell migration, and flow cytometry analysis of SHH signaling in pancreatic cancer cells (PCCs), *in vivo* and *in vitro* (Guo et al., 2021a). Quercetin showed an antitumor activity by downregulating c-Myc, leading to inhibition of PCC proliferation. Quercetin reduced the level of TGF-β1 and suppressed the epithelial-mesenchymal transition, thereby blocking migration and invasion. Quercetin induced apoptosis *via* death receptor pathways and the mitochondrial pathway. In an animal study in nude mice, treatment with quercetin reduced metastasis. The therapeutic effects of quercetin on PDA involve the regulation of SHH activity and are related to Gli2 but not Gli1. The quercetin-mediated suppression of PCC proliferation, migration, and invasion was abrogated when the SHH activity was increased using recombinant Shh protein. Moreover, by promoting the expression of Zeb2 and Snail1, Shh could activate TGF-β1/Smad2/3 signaling and promote EMT, resulting in a partial reversal of the quercetin-mediated suppression of PCC migration and invasion. These findings suggest that quercetin could be used to treat PDA by inhibiting PCC migration, metastasis, and invasion and by inducing apoptosis *via* antagonizing the TGF-β1/Smad and SHH signaling pathways (55).

Sarah Hassan and her colleagues evaluated the efficacy of a combination of quercetin with the known drugs gemcitabine (GEM) and doxorubicin (DOX) against human pancreatic cancer cells and hepatic cancer cells, respectively. They also evaluated the effects of quercetin on the activity of drug efflux pumps as well as the effects related to hypoxia. Moreover, they investigated the role of multidrug transporters (including MDR) and HIF-1α (Hassan et al., 2020). In their research, they combined quercetin with other anticancer drugs and showed that combination therapy (anticancer drug plus quercetin) had better results than single therapy. In 2D and 3D cultures, the percentage of dead cells increased to 60%. Deeper evaluation revealed that quercetin induced p53-mediated apoptosis *via* increased levels of the p53 regulator and also downregulated HIF-1α. Moreover quercetin could inhibit the MDR-1 efflux activity. In conclusion, they found quercetin could be administered in combination with GEM or DOX in multidrug resistant pancreatic and hepatic cancers, respectively (Hassan et al., 2020).

Liu et al. (2020) evaluated the effects of quercetin on GEM-resistant PC cells and its mechanism. They evaluated two cell lines from each of pancreatic and hepatocellular carcinoma. The PCC lines were BxPC-3 and PANC-1, and the HCC cell lines were HepG2 and Huh-7. Using a proliferation assay, it was found that quercetin had a cytotoxic effect on HepG2 and PANC-1 (GEM-resistant), and a flow cytometry analysis revealed a pro-apoptotic effect on HepG2 and PANC-1. Quercetin could induce apoptosis *via* upregulation of antitumor protein p53 and also cyclin-D1 downregulation, as seen by Western blotting. Moreover, it caused cell cycle arrest in the S phase. In conclusion, their data revealed that quercetin could be used in combination with known anticancer drugs against GEM-resistant hepatic and pancreatic cancers (Liu et al., 2020).

Hoca et al. (2020a) evaluated the effect of quercetin and resveratrol on the epithelial-mesenchymal transition (EMT) in pancreatic cancer cells (CD133-negative and CD133-positive). The CD133+ cancer stem cells were purified from PANC-1 cells using the MiniMACS system, and then the three cell variants (CD133+, CD133-, and PANC-1) received different doses of quercetin and resveratrol. Immunocytochemistry with antibodies against vimentin, TNF-α, ACTA-2, N-cadherin, IL-1β, and the MTT assay were employed. In the CD133 + cells that were treated with quercetin, the intensity of N-cadherin, ACTA-2, and IL-1β staining was reduced, compared to CD133 + cells treated with resveratrol. In conclusion, quercetin could reduce N-cadherin expression and prevent EMT and metastasis more than resveratrol in PCC stem cells (Hoca et al., 2020a). Table 1 lists some studies on the therapeutic effects of quercetin in pancreatic cancer.

**TABLE 1:** Studies on the therapeutic effects of quercetin in pancreatic cancer.

Type of Quercetin	Dose	Targets	Results	Model (*in vitro/in vivo/*Human)	Cell Line	Reference
Quercetin	100 µM	miR-142-3p, HSP70	Induced tumor cell death	*In vitro*	MIA PaCa-2, Capan-1, and HEK-293	MacKenzie et al. (2013)
Quercetin	50 μM	miR-200b-3p	Inhibited cancer stem cell self-renewal and proliferation	*In vitro*	AsPC1 and PANC1	Nwaeburu et al. (2017)
Quercetin	20 µM	hnRNPA1	Enhanced apoptosis, inhibited proliferation	*In vitro* and *in vivo*	K1 and 8505c	Pham et al. (2019)
Quercetin	5, 10, 25, 50, and 100 µM	ACTA-2, IL-1β, N-cadherin, TNF-α, and vimentin	Inhibited metastasis	*In vitro*	PANC-1 (ATCC: CRL-1469)	Hoca et al. (2019)
Quercetin	100 mg	β-catenin, vimentin, ZEB-1, caspase-3, and Bcl2	Enhanced the effect of anticancer drugs, sensitized cancer cells to chemotherapy and radiotherapy	*In vitro*	PANC-1, MIA PaCa-2, AsPC-1, and BxPC-3	Srivastava et al. (2011)
Quercetin	12.5 mg/kg	β-catenin	Induced cancer cell death	*In vitro* and *in vivo*	PANC1, MIAPaCa2, and BxPC3	Adikrisna et al. (2012)
Quercetin, dihydroquercetin	25–200 µM	Hsp70	Decreased cell viability and induced apoptosis	*In vitro* and *in vivo*	DMEM	Aghdassi et al. (2007)
Quercetin	1, 10, 50, and 100 μM	β-Catenin	Suppressed proliferation, invasion, self-renewal capacity, and CSC surface marker expression	*In vitro*	ASPC-1, BXPC-3, PANC-1, SW 1990, and HPAC	Cao et al. (2015)
Quercetin	50 and 10 μM	E-cadherin and Twist2	Induced apoptosis and reduced viability	*In vitro* and *in vivo*	BxPC-3/CRL-4023	Fan et al. (2016)
Quercetin-loaded chitosan nanoparticles	1 mg/ml		Significant toxicity for pancreatic cancer cells	*In vitro*	MIA PaCa2 and L929	David et al. (2015)
Quercetin	10, 100 μM		Significant toxicity for pancreatic cancer cells	*In vitro*	MIA PaCa2 and L929	David et al. (2015)
Quercetin	1,000 μg/ml	Caspase-3, -8, -9, cyclin-D1, -B1, and cyclin-dependent kinase 4	Induced both intrinsic and extrinsic apoptosis pathways and cell cycle arrest	*In vitro*	PANC-1 and CAPAN-1	Kim et al. (2019)
Quercetin	10.5; 0, 100, and 200 µM	HSP70	Increased apoptosis and autophagy	*In vitro*	Panc-1 and MIA PaCa-2	Hyun et al. (2013)
Quercetin	100, 200, and 400 μM	NF-κB	Anti-metastasis, inhibited proliferation, angiogenesis, and induced apoptosis	*In vitro* and *in vivo*	MIA PaCa2 and BxPC-3	Zhou et al. (2010)
Quercetin	200 mM	K-ras, miR-let-7, MMP-2, and ALDH1	Decreased viability, migration, and induced apoptosis	*In vitro*	BxPC-3, MIA PaCa2, and CRL-1097	Appari et al. (2014)
Quercetin	12.5, 25.50, and 100 μg/ml	Grp78/Bip and GADD153/CHOP	Induced apoptosis and increased effect against drug resistant pancreatic cancer cells	*In vitro*	PANC-1	Lee et al. (2013)
Quercetin	1.5, 1.9, and 2 μM	GSK-3b	Suppressed the growth of pancreatic tumors	*In vitro*	—	Johnson et al. (2011)
Quercetin	30, 60, and 90 μM	Caspase-8 and PARP	Induced apoptosis	*In vitro*	8988T	Kim et al. (2016)
Quercetin	6.25, 12.5, 25, and 50 μM	RAGE, PI3K, AKT, and mTOR	Regulated apoptosis and autophagy pathways and increased gemcitabine sensitivity	*In vitro*	MIA PaCa-2, BxPC-3, AsPC-1, HPAC, and PANC-1	Lan et al. (2019)
Quercetin, quercetin-3O-glucoside and quercetin-7O-glucoside	0, 200, 500, and 1,000 nM	EGFR	Anti-metastatic effect	*In vitro*	CFPAC-1, SNU-213, and PANC-1	Lee et al. (2015b)
Quercetin	100 μM	Caspase-3, cytochrome c, and NF-κB	Decreased primary tumor growth, increased apoptosis, and prevented metastasis	*In vitro* and *in vivo*	MIA PACA-229 and BSp73AS30	Mouria et al. (2002)
Quercetin	100, 0.2 µM	HSP70, caspase-3, and cytosolic cathepsin B	Induced apoptosis	*In vitro*	MIA PaCa-2 and PANC-1	Dudeja et al. (2009)
3′-O-methyl quercetin	30 µM	—	Inhibited tumor growth	*In vitro* and *in vivo*	MIA PaCa-2	Zhang et al. (2010)
Quercetin	50 and 100 μM	Fatty acid synthase (FAS)	Decreased tumor cell proliferation	*In vitro*	MIA PaCa-2	Harris et al. (2012)
Quercetin	20 μM	Bcl-2, XIAP, and caspase-3	Inhibited cell proliferation and induced apoptosis	*In vitro*	CD133+/CD44+/CD24+/ESA	Tang et al. (2012)
Quercetin	0–75 μM	Annexin V	Induced apoptosis and reduced proliferation	*In vitro* and *in vivo*	MIA PaCa-2 and BxPC-3	Angst et al. (2013)
Quercetin	50 μM	microRNA let-7c and Numbl	Decreased tumor growth	*In vitro* and *in vivo*	AsPC-1, CRL-4023, and PANC-1	Nwaeburu et al. (2016)
Quercetin	20, 40, 80, and 160 µM	MMP, STAT3, and IL-6	Inhibited EMT and decreased invasion and metastasis	*In vitro*	PANC-1 and PATU-8988	Yu et al. (2017)
Quercetin	10 μM	p53, K-Ras, PUMA, and p21	Induced p53 target genes, PUMA, and p21	*In vitro*	HCT116, A549, MKN-45, and MCF-7	Lee et al. (2009)
Quercetin	—	CD44	Inhibited migration of PCC	*In vitro*	MIA PaCa‐2 and PANC‐1	Serri et al. (2019)
Quercetin	1,535 μg/ml	—	Increased necrosis and late apoptosis in cancer cells	*In vitro*	PANC-1	Mohammed and Al-Omar (2021)
Quercetin	10 and 100 μM	SHH and TGF-β	Induced PCC apoptosis and reduced proliferation and metastasis	*In vitro*	PANC-1 and Patu 8,988	Guo et al. (2021b)
Quercetin	0–100 μM	HIF-1α and p53	Increased dead cells and increased apoptosis	*In vitro*	AsPC-1 and HepG2	Hassan et al. (2020)
Quercetin	0, 10, 25, 50, 100, and 200 μM	p53	S phase cell cycle arrest in GEM-resistant cells and downregulated cyclin D1	*In vitro*	BxPC-3, PANC-1, HepG2, and Huh-7	Liu et al. (2020)
Quercetin	50 μg/kg	Serotonin	Inhibited acinar-to-ductal metaplasia (ADM) and stem cell activation	*In vitro* and *in vivo*	—	Tao et al. (2020)
Quercetin	5–100 µM	TNF-α and vimentin	Inhibited metastasis	*In vitro*	CD133	Hoca et al. (2020b)

### 3.2 Quercetin and Gastric Cancer

Cing-Syuan Lei et al. tested whether a combination of quercetin and irinotecan might be effective for decreasing the metastasis of gastric cancer (GC) by measuring gene and protein expression (Lei et al., 2018). Their study compared the effect of low-dose SN-38 (irinotecan metabolite) in combination with quercetin with a high dose of SN-38 alone on β-catenin expression, cell viability, and apoptosis. *In vivo* xenograft animal models and *in vitro* studies looked at the effects of quercetin and low-dose irinotecan on GC metastasis. The β-catenin protein levels were lower in AGS cells treated with quercetin and a low dose of SN-38 than the single therapy using quercetin. ITG-β6 and Twist-1 gene expression (two EMT markers) as well as cyclooxygenase-2 gene expression were higher in high-dose irinotecan-treated cells than the combination therapy. In the AGS mouse model, VEGF-A (vascular endothelial growth factor), VEGF-receptor 2, and the percentage of Tie2-expressing monocytes were significantly lower after combined therapy. The data suggested that the treatment of GC with irinotecan could be improved by combining it with quercetin (Lei et al., 2018).

Hemati et al. evaluated the effect of si-RNA targeted against CDC20 (cell division cycle protein 20 homolog) and antiproliferative drugs (quercetin and DOX) against GC. They investigated niosome-encapsulated delivery vehicles for si-RNA and drugs. They found that si-RNA delivery in combination with anticancer drugs led to downregulation of CDC20, and therefore improved GC treatment (Hemati et al., 2019). To optimize the si-RNA loading capacity and physicochemical properties, they varied the cationic lipid content of cationic PEGylated niosomes. Quercetin and DOX as well as anti-CDC20 si-RNA were loaded into the co-delivery system, and physicochemical properties, controlled release, thermosensitivity, rates of apoptosis, and gene silencing efficacy were measured. Intriguingly, the data revealed that the co-delivery system, which was designed for loading si-RNA, had an appropriate high positive charge for drug delivery. They also showed a thermosensitive drug release behavior that successfully silenced CDC20 expression when compared with the single delivery of either si-RNA or the drug. Furthermore, their system effectively inhibited GC cell growth. Their data suggested that PEGylated niosomes co-loaded with CDC20 si-RNA plus anticancer drugs might be used as a novel system for GC treatment (Hemati et al., 2019).

Hai Li et al. evaluated the mechanism and effects of quercetin on metastasis of GC and also searched whether urokinase plasminogen activator and urokinase plasminogen receptor (uPA/uPAR) were involved in the mechanism or not. The uPA/uPAR system plays a key role in GC metastasis, so they planned the study to test whether quercetin could affect this system. In their study, they measured the amount of uPA and uPAR activity in precancerous tissues and compared them with different GC cell lines in terms of migration and invasion (Li and Chen 2018a). The data revealed that in precancerous tissues, uPA and uPAR activities were lower than that in GC cells, and the migration and invasion of GC cell lines were correlated with uPAR expression. uPA and uPAR protein expression levels were reduced along with migration and invasion after GC BGC823 and AGS cells were treated with quercetin (10 µM for 72 h). Quercetin combined with uPAR knockdown decreased matrix metalloproteinase-9 and metalloproteinase-2 activities, thereby inhibiting Pak1-Limk1-cofilin signaling. Quercetin treatment inhibited AMPKα activator, NF-κB, ERK1/2, and PKC-δ, which caused downregulation of uPA and uPAR expression. In conclusion, quercetin could be a novel component in GC therapy for reducing metastasis and invasion (Li and Chen 2018a).

Zeng et al. (2018) evaluated the effects of quercetin on GC. They treated human GC cells (NCI-N87) with 15 μM quercetin for 48 h along with dimethyl sulfoxide as a control. The HiSeq 2500 DNA sequence data were used to compare differentially expressed genes (DEGs) between groups. An advanced analysis was used to assess the protein–protein interaction (PPI) network. The regulatory network of transcription factors (TFs-DEGs) was elucidated using Cytoscape. The DEGS found were Fos proto-oncogene (FOS, degree = 12), aryl hydrocarbon receptor (AHR, degree = 12), Jun proto-oncogene (JUN, degree = 11), and cytochrome P450 family 1 subfamily A member 1 (CYP1A1, degree = 11), which were significantly associated with other proteins in the PPI network with higher degrees. Early growth response 1 (EGR1), FOS like 1 (FOSL1), FOS, and JUN were higher among the five TF-DEGs, whereas AHR was downregulated. The Wnt signaling pathway was also enriched for FOSL1, JUN, and Wnt family member 7B (WNT7B). In the PPI network, CYP1A1 was closely linked to AHR. Therefore, quercetin may have targeted FOS, AHR, JUN, CYP1A1, EGR1, FOSL1, and WNT7B in GC (Zeng et al., 2018).

Shang et al. (2018a) and his colleagues carried out *in vitro* studies on the potential of quercetin to induce human GC cell death, apoptosis, and alter gene expression. Their data revealed that quercetin could induce GC cell apoptosis and also change gene expression. Flow cytometry revealed that quercetin increased the level of reactive oxygen species (ROS) and led to the destruction of the mitochondrial membrane by reducing certain protein levels, and finally it caused apoptosis in AGS cells. The Western blotting showed that quercetin decreased the level of antiapoptotic proteins, including Bcl-x, Bcl-2, and Mcl-1, while increasing pro-apoptotic proteins, including Bid, Bax, and Bad. Quercetin induced various effects on gene expression. For instance, quercetin decreased the expression of KDELC2F (KDEL [Lys-Asp-Glu-Leu] containing 2), VEGF-B (vascular endothelial growth factor B), and CDK10 (cyclin-dependent kinase 10) but increased the expression of TP53INP1 (tumor protein p53 inducible nuclear protein 1), TNFRSF10D (tumor necrosis factor receptor superfamily 10D, decoy with truncated death domain), JUN-B (jun B proto-oncogene), and TP53INP1. In conclusion, their data revealed the molecular mechanism, gene expression, and signaling pathway involved in quercetin’s ability to inducing apoptosis in human GC cells (Shang et al., 2018a). Table 2 lists some studies on the therapeutic effects of quercetin in GC.

**TABLE 2:** Studies on the therapeutic effects of quercetin in gastric cancer.

Type of Quercetin	Dose	Targets	Results	Model (*in vitro/in vivo/*Human)	Cell Line	Reference
Quercetin	15 µM	FOS, AHR, CYP1A1, EGR1, FOSL1, and WNT7B	Antiproliferative effects	*In vitro*	NCI-N87	Zeng et al. (2018)
Quercetin	10–320 μM	Mcl-1, Bcl-2, Bcl-x, Bax, and MAPK	Induced apoptosis	*In vitro*	AGS	Shang et al. (2018b)
Quercetin	10 µM	NF-κb, PKC-δ, ERK1/2, and AMPKα	Inhibited expression of uPA, uPAR, and downstream targets	*In vitro*	BGC823 AGS	Li and Chen (2018b)
Quercetin	30 µM		Reduced the genotoxic effect of MNNG	*In vitro*	GMCs and PBLs	Arabski et al. (2006)
Quercetin	40 and 150 µM	GABARAPL1 and miR-143	Inhibited autophagy	*In vitro*	AGS/MNK28	Du et al. (2015)
Quercetin	15, 30, 60, 90, and 120 µM	Bcl-2, Bax, and caspase 3	Induced apoptosis	*In vitro*	BGC-823, MKN45, SW116, EC109, and Ges-1	Wang et al. (2012)
Quercetin	—	—	Regulated cell cycle and induced apoptosis	*In vitro*	MKN28	Shi et al. (2016)
Quercetin	3, 6, and 12 µM	ABCB1	Induced apoptosis and inhibited drug efflux	*In vitro*	EPG8-257RDB and EPG85-257P	Borska et al. (2012)
7-O-geranylquercetin	10, 15, and 20 µM	ROS-MAPK, P38, JNK, and ERK	Induced apoptosis and arrest cell cycle at G2/M phase	*In vitro*	SGC-7901, MGC-803, and GES-1	Zhu et al. (2017)
Quercetin-3-α-L-arabinofuranoside	10 μM, 1.25, 2.5, or 5 mg/kg	Bax, BOK, cleaved caspase-3, and PARP	Induced apoptosis and reduced tumor cell proliferation	*In vitro* and *in vivo*	SGC-7901, SGC-7901/DDP, and SGC-7901/5-Fu	Guo et al. (2018)
Quercetin	500 µM	MAPK and TRPM7	Induced apoptosis	*In vitro*	AGS and HEK293	Kim et al. (2014a)
Quercetin	20–100 µM	Bax, Bcl-2, cyt c, Oct4, Sox2, and CD44	Induced mitochondrial-dependent apoptosis	*In vitro*	MGC803	Shen et al. (2016)
Quercetin-loaded niosome	380 nm	CDC20-siRNA	Inhibited GC cell growth	*In vitro*	AGS	Hemati et al. (2019)
Quercetin	14–20 µM	CD74	Prevented *H. pylori* adhesion and subsequent infection	*In vitro*	NCI-N87 and Hs738St./Int	Sekiguchi et al. (2008)
Quercetin	6.25, 12.5, 25, 50, and 100 mg/kg	VEGF-A, VEGF-A receptor 2, and Tie2	Enhanced the efficacy of irinotecan/SN-38	*In vitro* and *in vivo*	AGS	Lei et al. (2018)
3-O-methylquercetin	1 mg/kg	NF-κB	Decreased viability and expression of proliferative and angiogenic biomarkers and induced apoptosis	*In vitro* and *in vivo*	AGS, SNU-5,SNU-16, MKN45, NUGC3, and AZ521	Manu et al. (2015)
Quercetin	10 µM	V-FITC/PI, cyt c, ERK, and AKT	Inhibited proliferation and induced apoptosis	*In vitro*	MGC-803	Zhang et al. (2015a)
Quercetin	8–1,024 μg/ml	p38MAPK, Bcl-2, and BAX	Regulated the balance of proliferation and apoptosis	*In vitro* and *in vivo*	GES-1	Zhang et al. (2017a)
Quercetin	40 and 160 μM	mTOR1, Beclin1, and Bcl-2	Induced protective autophagy	*In vitro* and *in vivo*	AGS and MKN28	Wang et al. (2011)
Quercetin	70 µM	DNA and RNA	Blocked cell cycle progression	*In vitro*	HGC-27, NUGC-2, MKN7, and MKN28	Yoshida et al. (1990)
3ˊ-O-methylated quercetin	10–50 µM	PPARγ	Induced apoptosis	*In vitro* and *in vivo*	AGS	Ramachandran et al. (2012)
Quercetin	29.2–40.3 mM	pSTAT3	Inhibited survivin expression and reduced viability	*In vitro*	AGS	Pandey et al. (2015)
Quercetin	12.5 μg/ml	Bax/Bcl-2, and caspase-8	Induced apoptosis	*In vitro*	MGC80-3	Zhou et al. (2019b)

### 3.3 Quercetin and Colorectal Cancer

Mostafa et al. carried out a study to design and synthesize multi-target anticancer drugs for CRC treatment. Their agents targeted some essential enzymes for CRC survival and proliferation, including PIM-1, 5-LOX, and COX-2 (117). Compounds **5a-d** and **5g** inhibited the COX-2 enzyme similar to celecoxib with a high selectivity index. Compound **5b-e** inhibited the 5-LOX enzyme to the same extent as quercetin, while 5g, 5f, and 5a showed slightly lower inhibitory activity than quercetin. They concluded that **5e-g** and **5a-c** were better than Celecoxib, while 5g, 5f, and 5a were better than diclofenac sodium, in an *in vivo* model. Compounds 5e-g and 5a-c showed a better safety profile than celecoxib in fasted rats. Compounds 5g, 5f, and 5d had the highest potency against CRC cell lines (HCT-116 and Caco-2) at a much lower dosage than against normal human cells. Moreover, compounds 5g and 5e induced apoptosis in CRC cell lines and induced caspase activation. Compounds 5d, 5g, and 5e inhibited PIM1 and PIM2 kinase, which was comparable to the reference staurosporine (El-Miligy et al., 2021).

Ghamedi et al. evaluated the mechanism and effects of a 200 mg dose of quercetin and a 150 mg dose of EGCG (epigallocatechin gallate) at varying proportions on the induction of apoptosis and inhibition of proliferation of human CRC cell line HCT-116 (Al-Ghamdi et al., 2021). The phytomolecules inhibited cell growth, arrested cell cycle, annexin V, and reduced clonogenicity. Colony formation was inhibited by the lowest dose of the tested drugs. Furthermore, a significant increase in annexin V was found at 150 mg dose of quercetin and 100 mg dose of EGCG. The combination therapy induced cell cycle arrest at the G1 phase. In conclusion, the combination of EGCG plus quercetin could be used as effective combination chemotherapy in the future, but more studies are required to establish a suitable dose and any side effects (Al-Ghamdi et al., 2021).

Erdoğan et al. (2021) evaluated the antiangiogenic effects of quercetin and luteolin on colon cancer cells (HT29) and also their anticancer effects compared to the traditional chemotherapy drug 5FU, as well as a combination of 5FU with luteolin or quercetin. They used Western blotting, qRT-PCR, human VEGF ELISA, fluorescence microscopy, and an MTT assay. They used Western blotting to assess the effects on the genes p53, Bax, Bcl-2, P38, MAPK, PTEN, Akt protein, and mTOR. ELISA was used to determine the effects of treatment on angiogenesis and the MTT assay to measure cell viability. Fluorescence microscopy was used to detect apoptosis in HT29 cells. The induction of apoptosis in cells treated with quercetin and 5FU was eight times higher than the control, and it was 10 times higher for luteolin and 5FU. The VEGF level was significantly lower in cells treated with a combination of quercetin or luteolin plus 5FU. They found that quercetin and luteolin could regulate apoptosis in HT-29 cells and also that combination therapy reduced antiapoptotic factors, including Bcl-2, mTOR, and Akt gene expression compared to the control group. P53, P38, MPK, and PTEN gene expression increased faster in groups treated with 5FU and quercetin than in cells treated with 5FU and luteolin. In conclusion, luteolin and quercetin could synergistically increase the anticancer effect of 5FU and could also reduce the toxic effects of 5FU in colorectal cancer (Erdoğan et al., 2021).

In a study by Wenhui Liu et al., they induced constipation (a known colorectal risk factor) by administering loperamid in a rat model, and then evaluated the effects of quercetin on loperamid-induced constipation (Liu and Zhi 2021). The data revealed that in rats that were treated with 25 mg/kg and 50 mg/kg of quercetin, the intestinal transit rate was increased, as well as the concentration of short-chain fatty acids and levels of gastrin, motilin, and substance P. Moreover, quercetin improved intestinal peristalsis movement and reduced somatostatin levels. The expression of aquaporin 3, transient receptor potential vanilloid 1, glial cell line-derived neurotrophic factor, enteric nerve-related factors, nitric oxide synthase, stem cell factor, and c-Kit was evaluated by Western blotting and RT-qPCR. They found that quercetin treatment reduced loperamid-induced constipation by increasing the markers of interstitial cells of Cajal, including stem cell factor, its receptor c-Kit, and AQP3. In conclusion, their data showed that quercetin had protective effects against loperamide-induced constipation, so it could be used to reduce the risk of colorectal cancer (Liu and Zhi 2021).

Iván Benito et al. evaluated the effects of daily supplementation with microencapsulated probiotics alone or in combination with microencapsulated quercetin, for the prevention of colorectal cancer. They used ApcMin/+ mice which spontaneously develop intestinal adenomas and carcinoma. They assessed histological alterations, intestinal bleeding, fat depots, respiratory quotient, body weight, and energy expenditure. Furthermore, they evaluated gene expression involved in the Wnt signaling pathway (Benito et al., 2021). ApcMin/+ mice were administered with Bf and Lg probiotic strains, 10 (7) CFU/100 g of food, or both probiotic strains plus microencapsulated quercetin (15 mg/100 g of food) for 73 days. Then they evaluated the energy metabolism, changes in organ and body weight, colon tissue histology, intestinal microbiota, and Wnt signaling pathway gene expression. The data revealed that the microencapsulated supplement (probiotics plus quercetin) could prevent colorectal cancer progression in ApcMin/+ mice (Benito et al., 2021).

Shree et al. (2021) evaluated the role of quercetin in apoptosis, hyperproliferation, and inflammation and also its mechanism in 1,2-dimethylhydrazine (DMH)-induced carcinogenicity and tumor multiplicity. They administered the rats with quercetin at 25 or 50 mg/kg body weight orally and 20 mg/kg bodyweight of DMH subcutaneously for 15 weeks and then sacrificed them. The DMH produces reactive oxygen species (superoxide) by membrane lipid peroxidation and causes an imbalance in redox homeostasis. DMH also decreases tissue antioxidant levels. Proliferative and inflammatory factors were increased in DMH-induced intestinal carcinogenicity as a result of low Bax/Bcl-2 ratio and apoptosis dysregulation. Quercetin pretreatment reduced the harmful effects of DMH, including preserving the detoxifying enzyme activity and reducing proliferation and early markers (mucin depletion and goblet cell disintegration) in colonic tissue. Quercetin regulated the expression of β-catenin and APC and lowered the incidence and multiplicity of tumors. The histological results further confirmed the beneficial role of quercetin in reducing DMH-induced pathological alterations (Shree et al., 2021). Table 3 lists some studies on the therapeutic effect of quercetin in colorectal cancer.

**TABLE 3:** Studies on the therapeutic effects of quercetin in colorectal cancer.

Type of Quercetin	Dose	Targets	Results	Model (*in vitro/in vivo/*Human)	Cell Line	Refrence
Quercetin	50 mg/kg	TAG72, GAL3, Wnt5a, colon and Axin-1	Induced apoptosis	*In vivo*	—	Ahmed et al. (2016)
Quercetin	4.5 g/kg	COX-1, COX-2, iNOS	Antiproliferation and induced apoptosis	*In vivo*	—	Warren et al. (2009)
Quercetin	50 g/kg	—	Fewer ACF	*In vivo*	—	Gee et al. (2002)
Quercetin	5 µM	MMP-2, MMP-9, E-cadherin, TNF-α, COX-2, and IL-6	Anti-metastatic and anti-invasion	*In vitro*	Caco-2	Han et al. (2016)
Quercetin	12 µM	p53, mitochondrial apoptosis pathway, and siRNA	Increased the cytotoxicity and apoptosis of 5-FU	*In vitro*	CO-115 and HCT-15	Xavier et al. (2011)
Quercetin	50 µM	NF-κB	Induced apoptosis	*In vitro*	HT-29	Jin et al. (2016)
Quercetin	75 µM	G2/M	Antiproliferation and induced apoptosis	*In vitro*	HT-29	Atashpour et al. (2015)
Quercetin	0–200 µM	Akt, p53, Bcl-2	Induced apoptosis	*In vitro*	HT-29	Yang et al. (2016)
Quercetin	25 and 50 µM	Sestrin 2, AMPK, mTOR, and ROS	Induced apoptosis	*In vitro*	HCT-116	Kim et al. (2013)
Quercetin	25 and 50 µM	MAPK, sestrin 2, and ROS	Induced apoptosis	*In vitro*	HT-29	Kim et al. (2014b)
Quercetin	20 and 15 μM	MAPK and PI3 K	Antiproliferation and induced apoptosis	*In vitro*	HCT-15 and CO-115	Xavier et al. (2009)
Quercetin	100 μM	ErbB-2, ErbB-3, AKT, and caspase 3	Induced apoptosis	*In vitro*	HT-29	Kim et al. (2005)
Quercetin	50 and 100 µM	COX-2 and IκBα	Anti-inflammatory	*In vitro*	HT-29	Narayansingh and Hurta (2009)
Quercetin	200 µM	NF-κB	Induced apoptosis	*In vitro*	Caco-2 and SW-620	Zhang et al. (2015b)
Quercetin	50 µM	Wnt, β-catenin	Downregulated β-catenin, Tcf signaling	*In vitro*	SW-480	Park et al. (2005)
Quercetin	15 µM	ERK	Induced autophagy	*In vitro*	SW-620 and HCT-116	Zhao et al. (2017)
Quercetin	20 μM	RAS genes	Induced autophagy and reduced viability	*In vitro*	Caco-2	Psahoulia et al. (2007a)
Quercetin	10nM–10 µM	Type-II EBS	Antiproliferation	*In vitro*	HT-29, COLO 20 I, and LS- I74T	Ranelletti et al. (1992)
Quercetin	10, 20, and 50 µM	CB1-R, PI3K, and JNK/JUN	Antiproliferation and induced apoptosis	*In vitro*	Caco-2, DLD-1	Refolo et al. (2015)
Quercetin	7 µM	CAMP	Cytotoxic to cancer cells	*In vitro*	HT-29	Agullo et al. (1996)
Quercetin	≥50 µM	Ornithine decarboxylase	Induced apoptosis	*In vitro*	DLD-1	Linsalata et al. (2010)
Quercetin	15–120 µM	—	Cytotoxic and antiproliferation	*In vitro*	HT-29 and Caco-2	Agullo et al. (1994)
Quercetin	100 µM	*p21*, *CDKN2B, TNFSF15*, *RGS5*,SMAD4, SESN2, and VEGF	Induced apoptosis	*In vitro*	CO-115	Murtaza et al. (2006)
Quercetin	30–40 µM	—	Induced apoptosis	*In vitro*	HT-29 and Caco-2	Kuo (1996)
Quercetin	100 µM	Caspase-3	Induced apoptosis	*In vitro*	HT-29 and Caco-2	Kuntz et al. (1999)
Quercetin-50 and 8-di-sulfonate sodium (QS)	100 µM	ROS	Induced apoptosis	*In vitro*	LoVo	Zhang et al. (2012)
Quercetin	150 µM	Caspase-3	Induced apoptosis	*In vitro*	HT-29	Wenzel et al. (2004)
Quercetin	30 µM	TRAIL	Induced apoptosis	*In vitro*	HT-29, SW-620, Caco-2	Psahoulia et al. (2007b)
Quercetin	0–40 µM	NAG-1 siRNA, EGR-1, and p53	Induced apoptosis	*In vitro*	HCT-116	Lim et al. (2007)
Quercetin	0.1–1 µM	ERβ and PTEN	Induced apoptosis	*In vitro*	DLD-1	Bulzomi et al. (2012)
Quercetin	50 mg/kg	AMPK and HIF-1	Induced apoptosis	*In vitro* and *in vivo*	HCT-116	Kim et al. (2012)
Quercetin	100 or 200 µM	AMP kinase	Induced apoptosis	*In vitro*	HT-29	Lee et al. (2010)
Quercetin	100 µM	AMPK and p53	Induced apoptosis	*In vitro*	HT-29	Kim et al. (2010)
Quercetin	30 and 80 µM	—	Antiproliferative effect	*In vitro*	HCT-116 and HT-29	van der Woude et al. (2003)
Quercetin	0–80 µM	β-catenin	Reduced colorectal carcinogenesis	*In vitro* and *in vivo*	Caco-2 and Fisher 344 rats	Dihal et al. (2006)
Quercetin	5 µM	Antigen Ki67	Antiproliferative effect	*In vitro*	HuTu-80 and Caco2	Ackland et al. (2005)
Quercetin	17.5 µM	17 kDa protein	Antiproliferative effect and inhibited cancer cell growth	*In vitro*	COL0320 DM	Hosokawa et al. (1990)
Quercetin	160 µM	Wnt	G1/S phase cell cycle arrest	*In vitro*	SW-480	Shan et al. (2009)
Quercetin	5 and 50 μM	CDC6, CDK4, and cyclin D1	Inhibited cell cycle	*In vitro*	Caco-2	van Erk et al. (2005)
Quercetin	200 mg/kg	TNF-α, Hmgcs2, Fabp2, and Gpt	Anti-inflammatory	*In vivo*	—	Qi et al. (2019)
Quercetin	100 mg	EGFR, Akt, Cdk1, cyclin B, and VEGF	Induced apoptosis, antiangiogenesis, and antiproliferation	*In vivo*	—	Rashedi et al. (2019)
Quercetin	100–500 µg/ml	ROS	Antiproliferation	*In vitro*	*Caco-2*	Zhou et al. (2019c)
Quercetin	648 μg/ml	TNF-a and TNF-R1	Induced apoptosis and antioxidant	*In vitro*	HCT116	Sezer et al. (2019)
Quercetin	100 mg/kg	HDAC8 and caspase 3/7	Induced apoptosis	*In vitro* and *in vivo*	HCT116	Biswas and Reddy (2018)
Quercetin	30 μM	Nrf-2 and Prx-6 protein	Inhibited lipid peroxidation	*In vitro*	*Caco-2*	Morales et al. (2018)
Quercetin	100 µM	KRAS, JNK, and caspase-3	Induced apoptosis	*In vitro*	DLD-1	Yang et al. (2019b)
Quercetin	50–200 µM	Akt, p53, and Bcl-2	Induced apoptosis	*In vitro*	HT-29	Velázquez et al. (2014)

In a study by Antara Banerjee et al., the effects of a combination of *Lycopodium clavatum* and quercetin on CRC cells (Colo-320) were evaluated by measuring expression of extracellular matrix proteins, cytotoxicity assay, morphological alterations, and expression of apoptotic genes. Furthermore, the anti-inflammatory, apoptotic, and proliferative responses of cancer cells treated with *Lycopodium* and quercetin alone or in combination, were evaluated to assess potential synergistic effects and expression of tumor suppressor genes (Banerjee et al., 2020). Their study was mainly conducted to identify whether *L. clavatum* extract or quercetin or a combination could produce significant anti-inflammatory effects in Colo-320 cells. Gelatin zymmography, toxicity biomarkers, and apoptotic gene expression were measured. The combination of quercetin (50 μm) and *L. clavatum* extract (10 μL) significantly attenuated cell growth and decreased the proliferation potential and colony formation compared to their separate administration. Antimicrobial assays showed that *Lycopodium* exerted antimicrobial activity against *Pseudomonas aeruginosa* and *Escherichia coli*. The quercetin and *L. clavatum* extracts contain flavonoids and alkaloids. Gelatin zymography showed a prominent decrease in MMP9 and MMP2 activity following administration of the quercetin and Lycopodium combination. This regimen altered the expression of catalase, cyclin D1, Bcl2, caspase-3, Bax, and Wnt1 genes in colon cancer cells. Quercetin and *Lycopodium* showed synergistic effects which may effectively attenuate progression of CRC with few adverse effects and no drug resistance (Banerjee et al., 2020).

Yana Li et al. evaluated whether quercetin could sensitize colon cancer cells to radiotherapy, considering the fact that quercetin can inhibit the Notch-1 signaling pathway and thus reduce proliferation of colon cancer cells and CSCs (colon cancer stem cells) (Li et al., 2020). Their study showed that a combination of quercetin with ionizing radiation (IR) had more significant antitumor effects than either IR or quercetin alone by suppressing the Notch-1 signaling pathway in CSCs. These findings were further validated using an *in vivo* xenograft of human colon cancer in nude mice, which showed a significant reduction in CSC markers and Notch-1 signaling proteins. Combined treatment with low-dose IR and quercetin remarkably decreased the expression of all gamma-secretase complex proteins in DLD-1 and HT-29 cells. Moreover, the combination therapy was partly abrogated by altering the expression of NICD (Notch intracellular domain). Finally, their study suggested that a combination of IR (5 Gy) and quercetin (20 mM) could be a novel therapeutic approach in colon cancer by suppressing the Notch-1 signaling pathway in CSCs. Further studies are required to confirm the therapeutic potential of combined quercetin–radiotherapy in clinical trials (Li et al., 2020).

### 3.4 Quercetin and Esophageal Cancer

In a study by Xin et al., it was shown that NF-κB plays a major role in resistance to chemotherapy in esophageal cancer cells treated with 5-FU. Their study mainly focused on the potential benefits of quercetin on the chemosensitivity of human esophageal cancer and identifying the underlying antitumor mechanism (Chuang-Xin et al., 2012a). Their study evaluated the effect of combination of quercetin and traditional chemotherapeutic drugs on esophageal carcinoma. The MMT assay was employed to measure the effects of quercetin on Eca109 and EC9706 cell proliferation. Western blotting was employed to evaluate protein levels, while apoptosis was investigated using FACS (Annexin V-FITC/propidium iodide). Their study showed that the combination of 5-FU and quercetin considerably reduced cell growth and provoked apoptosis in esophageal cancer cell lines (Eca109 and EC9706) in comparison with either 5-FU or quercetin alone. These effects were correlated with the downregulation of phosphorylated inhibitor of NF-κB (pIκBα) in response to treatment with 5-FU alone. In conclusion, the addition of quercetin to the conventional chemotherapeutic drug 5-FU may improve treatment of esophageal cancer (Chuang-Xin et al., 2012a).

Zhao et al. investigated whether quercetin-3-methyl ether (Q3ME), which is a flavonoid frequently found in tea, vegetables, fruits, wine, and tea, could inhibit the formation and progression of preneoplastic esophageal lesions produced by N-nitrosomethylbenzylamine. They studied inflammation and proliferation of esophageal cells *in vivo*. Q3ME suppressed esophageal cancer cell proliferation as well as the malignant transformation of healthy esophageal cells by inhibiting the MAPK and AKT/mTOR/p70S6K signaling pathways. Q3ME inhibited esophageal tumorigenesis by targeting ERKs and AKT (Zhao et al., 2018a). They found that phosphorylated ERKs (p-ERKs) and p-AKT were overexpressed in esophageal cancer cell lines and in tissue samples from patients with esophageal cancer. Using a human pull-down assay and a phosphokinase array, they showed that Q3ME could interact with ERKs and AKT and inhibit their kinase activity. Mechanistically, Q3ME reduced proliferation of esophageal cancer cells and anchorage-independent growth. According to Western blotting, Q3ME could inhibit the activity of ERK and AKT downstream signaling pathways and subsequently suppress AP-1 (activating protein-1) transcription factor. Interestingly, Q3ME also suppressed the development of preneoplastic lesions caused by NMBA. This inhibitory effect was correlated with attenuated proliferation of esophageal cancer cells and lower inflammation *in vivo* (Zhao et al., 2018a).

Yue Liu et al. evaluated the effects of quercetin on the angiogenesis and migration of esophageal cancer cells, in addition to the underlying mechanism (Liu et al., 2021). In their study, human esophageal cancer cells (Eca109) received 5 or 10 μg/ml of quercetin. A scratch wound healing assay evaluated cell migration, invasion was examined using a transwell assay, and a colony formation assay was conducted. Human umbilical vein vascular endothelial cells (CLR-1730) were inoculated in Eca109 conditioned medium, and the effects of quercetin were measured by tube formation and wound healing assays. Western blotting measured the levels of MMP2, MMP9, and VEGF-A protein expression. Quercetin (10 μg/ml) decreased colony formation in Eca109 cells; however, no difference was observed between the control group and the 5 μg/ml quercetin group. The group treated with 10 μg/ml quercetin showed reduced cell migration and invasion, while 5 μg/ml only suppressed invasion. Tube formation ability and migration of endothelial cells was inhibited in cells incubated in Eca109 conditioned medium. The group treated with 10 μg/ml quercetin showed reduced levels of MMP2, MMP9, and VEGF-A (Liu et al., 2021). Table 4 lists some studies on the therapeutic effects of quercetin in esophageal cancer.

**TABLE 4:** Studies on the therapeutic effects of quercetin in esophageal cancer.

Type of Quercetin	Dose	Targets	Results	Model (*in vitro/in vivo/*Human)	Cell Line	Reference
Quercetin-3-methyl ether	0–10 μM	AKT/mTOR/p70S6K, and MAPK	Anti-inflammatory, antiproliferation, and inhibited tumor growth	*In vitro*	SHEE and KYSE450	Zhao et al. (2018b)
		ERK, Ki67, c-Jun, and p-p70S6K		*In vivo*	KYSE510	
Liposomal/nanoliposomal quercetin	40 μmol	HDAC1, NF-κB, Cyclin D1, and caspase-3	Induced apoptosis	*In vitro*	Eca109	Zheng et al. (2014a)
					Eca9706	
Quercetin	20, 40, and 60 μM	DNMT1, NF-κB, HDAC1, cyclin D1, and caspase-3	Induced apoptosis	*In vitro*	Eca9706	Zheng et al. (2014b)
Quercetin	12.5–200	NF-κB	Antiproliferation and induced apoptosis	*In vitro*	EC9706 Eca109	Chuang-Xin et al. (2012b)
	µM					
Quercetin	10–80 µM	p21, cyclin B1, and caspase 3.9	Induced apoptosis and G2/M cell cycle arrest	*In vitro*	KYSE-510	Zhang et al. (2009b)
Quercetin	10–80 µM	PIG3, cyclin B1, caspase-3, and caspase-9	Induced apoptosis	*In vitro*	OE33	Zhang et al. (2008b)
			G2/M cell cycle arrest			
Quercetin	0–50 µM	COX-2 and PGE-2	Induced apoptosis and cell cycle arrest	*In vitro*	OE33	Cheong et al. (2004)
Quercetin	10 μg/ml	VEGF-A, MMP2, and MMP9	Suppressed invasion and angiogenesis of esophageal cancer cells	*In vitro*	Eca109	Liu et al. (2021)

### 3.5 Quercetin and Hepatocellular Carcinoma

Yamada et al., reported that myricetin and quercetin could suppress the AKT signaling axis, which may subsequently inhibit TGF-α- and HGF-mediated HuH7 cell migration (Yamada et al., 2020). In their study, the effects of quercetin on the migration of HuH7 cancer cells induced by TGF-α or HGF were investigated. Quercetin prominently inhibited the migration of HuH7 cells mediated by both TGF-α and HGF in a dose-dependent manner. Furthermore, myricetin (another flavonol compound) also prominently suppressed the migration of cancer cells. Neither myricetin nor quercetin affected the autophosphorylation of receptors mediated by TGF-α and HGF. Moreover, quercetin did not affect the phosphorylation of p38 MAPK induced by TGF-α or HGF. On the other hand, both myricetin and quercetin suppressed AKT phosphorylation mediated by the growth factors. Their study showed that quercetin could inhibit HCC cell migration mediated by growth factors by blocking the AKT signaling pathway but not p38 MAPK (Yamada et al., 2020).

Zhao et al., conducted a study to evaluate whether a combination of quercetin plus cisplatin could produce synergistic effects in HCC cells (Zhao et al., 2014). The hepatocellular carcinoma cell line HepG2 was treated with either cisplatin (10 μM) or quercetin (50 μM) or their combination, and apoptosis and cell proliferation were measured. The cisplatin and quercetin combination triggered apoptosis and inhibited proliferation in HepG2 cells compared with either agent alone. The combination of cisplatin and quercetin affected the expression of genes involved in the cell cycle and apoptosis. Administration of quercetin alone led to a significant increase in p16 expression in HepG2 cells. G1 phase cell cycle arrest induced by quercetin and apoptosis in HepG2 cells were partly abrogated by p16 targeted knockdown using RNA interference technology. Therefore, the cell cycle arrest and apoptosis caused by quercetin plus cisplatin likely involved p16 and provided a more prominent antiproliferative and apoptotic effect (Zhao et al., 2014).

Ding and colleagues showed that quercetin could inhibit the proteasome activity and decrease the phosphorylation of ERK1/2. The increased activity of ERK1/2 led to an increase in the proteasome chymotrypsin-like activity, whereas the increased activity of MEK1 resulted in a lower proteasome chymotrypsin-like activity. Administration of quercetin mitigated the expression of proteasome β subunits. Their study showed that the MEK1/ERK1/2 signaling pathway could modulate the expression of proteasome β subunits, leading to a lower proteasome chymotrypsin-like activity (Ding et al., 2018). Caspase and trypsin-like protease activities remained constant in HepG2 cells along with an increased activity of JNK and p38 MAPK and attenuated phosphorylation of ERK1/2. The decreased proteasome activity induced by quercetin may not be reversed following suppression of the JNK and p38 MAPK signaling pathways. Upregulation or downregulation of MEK1 enhanced or attenuated proteasome chymotrypsin-like activity, respectively. The expression of β subunits of proteasome was reduced by both MEK1/ERK1/2 inhibition and by quercetin administration (Ding et al., 2018).

Srisa-Nga et al. designed a study to prepare quercetin magnetic nanocarriers using polymeric micelles to allow monitoring and treatment of hepatocellular carcinoma. These polymeric micelles were characterized based on their morphology, size, and magnetic properties. They evaluated the cellular uptake, cytotoxicity, cell cycle analysis, and potential magnetic targeting in HCC cells transfected with hepatitis B virus (HepG2.2.15) (Srisa-Nga et al., 2019). They co-encapsulated superparamagnetic iron oxide nanoparticles (SPIONs) and quercetin (QCT) into mPEG750-b-OCL-Bz micelles (QCT-SPION micelles) and added methoxy-poly (ethylene glycol)-b-oligo-3-caprolactone in order to inhibit the growth of HepG2.2.15 cells. The QCT-SPION micelles were prepared using the film hydration technique. They had a spherical morphology with an average size of 22–55 nm. The optimized micelles showed a quercetin loading capacity and entrapment efficiency of 3.5 and 70%, respectively. The SPIONs alone had a T2 relaxivity of 137 mM^−1^ s^−1^, while the cluster of SPIONs inside the SPION-QCT micelles had a T2 relaxivity value of about 246 mM^−1^ s^−1^, implying a higher sensitivity for magnetic resonance imaging. The SPION-QCT micelles were taken up by HepG2.2.15 cells and showed more cytotoxicity than quercetin, along with cell cycle arrest at the G0/G1 phase. The SPION-QCT micelles accumulated in the vicinity of neodymium–iron–boron (NdFeB) magnetic discs, and the application of a strong magnetic field further inhibited tumor cell growth. These mPEG750-b-OCL-Bz micelles acted as a novel multifunctional co-delivery vehicle for SPIONs and quercetin for monitoring and treatment of HCC (Srisa-Nga et al., 2019).

Pasachan et al. designed a study to evaluate the antidiabetic effects of TTE (*Tiliacora triandra* Colebr) on the gluconeogenesis of liver cells and HCC cells (HepG2). The principal constituents of TTE include quercetin and epicatechin. They investigated the underlying mechanism and the *in vivo* effects in a type 2 diabetes mellitus (T2DM) rat model. They asked whether TTE could act as a nutritional agent in the management of T2DM and prevent complications such as non-alcoholic steatohepatitis, non-alcoholic fatty liver disease, and eventual cirrhosis (Pasachan et al., 2021a). They used a Diels aqueous extract of TTE to affect glucose synthesis in cells with T2DM and HepG2 cells. The HepG2 cells received TTE and purified quercetin and epicatechin. Hepatic gluconeogenesis was evaluated in rats with T2DM, which received either daily TTE (1,000 mg/kg body weight), metformin (30 mg/kg), or the combination of metformin plus TTE over a period of 12 weeks. Similar to quercetin and epicatechin, TTE stimulated gene expression of catalase, glutathione peroxidase, and copper–zinc superoxide dismutase. TTE lowered the synthesis of new glucose by suppressing phosphoenolpyruvate carboxykinase and glucose-6-phosphatase and stimulating AMP-induced protein kinase phosphorylation in HepG2 cells. Similar to metformin, TTE showed antidiabetic, anti-triglyceridemic, anti-hyperglycemic, and antioxidant effects in rats with T2DM. TTE may be used as a nutraceutical agent in individuals with insulin resistance, obesity, or those receiving antidiabetic medication (Pasachan et al., 2021a). Table 5 lists some studies on the therapeutic effects of quercetin in HCC.

**TABLE 5:** Studies on the therapeutic effects of quercetin in HCC.

Type of Quercetin	Dose	Targets	Results	Model (*in vitro/in vivo/*Human)	Cell Line	Reference
Quercetin	100 mg/kg	CK2α, Notch1, Gli2, caspase-3, p53, cyclin-D1, and Ki-67	Antiproliferation, antioxidant, and antiapoptosis	*In vivo*	—	Salama et al. (2019)
Quercetin	100–300 μg/ml	—	Prevented CCl4-induced cytotoxicity	*In vitro*	HepG2	Vijayakumar et al. (2019)
Quercetin	0–200 µM	JAK2 and STAT3	Antiproliferation, cell cycle arrest, induced apoptosis, anti-migration, and anti-invasion	*In vitro* and *in vivo*	LM3	Wu et al. (2019a)
QRC/SPC co-loaded NCs	0–100 µM	kappa B, TNF-α, and Ki-67	Enhancing SFB antitumor efficacy.(antiproliferative and anti-vascularization)	*In vivo* and *In vitro*	HepG2	Abdelmoneem et al. (2019)
Quercetin	12.5–50 µM	Hexokinase-2 and AKT/mTOR	Antiproliferative effect	*In vitro* and *In vivo*	SMMG-7721 and BEL-7402	Wu et al. (2019b)
Quercetin	0–80 μM	AKT/mTOR and MAPK	Autophagy stimulation and Induced apoptosis	*In vitro*	MMC7721	Ji et al. (2019)
	*In vivo*			HepG2		
Quercetin	0, 20, 40, and 80 µM	Intracellular ROS, p53	Antiproliferative effect	*In vitro*	HepG2	Jeon et al. (2019)
Ziziphus spina-christi (ZSCL)	100 and 300 mg/kg	Hepatocyte growth factor	Antioxidant effects and anti-oncogenic effects	*In vivo*	HepG2	El-Din et al. (2019)
	Insulin-like growth factor-1 receptor	*In vitro*				
Quercetin, dasatinib	5, 50 mg/kg	SASP, P16, and γH2AX foci	Pro-tumorigenic effects	*In vivo*	HepG2 and Huh-7	Kovacovicova et al. (2018)
	*In vitro*					
QCT-SPION-loaded micelles	0–60 µM	—	Increased cytotoxicity, cell cycle arrest, and antiproliferation	*In vitro*	HepG2.2.15	Srisa-Nga et al. (2019)
Quercetin	20–160 μM	Cyclin A, B2, D1, Bcl-2, caspase-3, and -9	Antiproliferation and induced apoptosis	*In vitro*	Hep3b and HepG2	Bahman et al. (2018)
Nanocarriers of quercetin	1, 550, and 150 µM	Caspase-3, H2O2, c-MET, and MCL-1	Induced apoptosis	*In vitro*	HepG2 and HeLa	AbouAitah et al. (2018)
Quercetin	40, 80, and 160 μM	ABCB1, ABCC1, ABCC2, and Wnt	Enhanced sensitivity and increased cellular accumulation of chemotherapy drugs	*In vitro*	BEL/5-FU	Chen et al. (2018)
	BEL-7402					
Quercetin (SFJDC)	6.75 μg/ml	Bcl-2, Bax, Akt/mTOR, and NF-κB	Induced apoptosis, inhibited migration and invasion, affected, af	*In vitro*	HepG2 HepG2.2.15	Xia et al. (2018)
Quercetin	0–100 μM	p38, MAPK, JNK, and MEK1	Induced apoptosis	*In vitro*	HepG2	Ding et al. (2018)
Quercetin	5–50 μM	NF-κB	Enhanced Antiproliferative effects and induced apoptosis	*In vitro*	SMMC-7721	Zou et al. (2018)
	*In vivo*			HepG2, HuH-7		
Quercetin	10, 25, and 50 μΜ	JAK, SHP2 phosphatase, and IFN-α	Antiproliferative effect	*In vitro*	HepG2 Huh7	Igbe et al. (2017)
3′,4′,7-Tri-O quercetin	25 mg	—	Stability indicator for hydrolytic degradation	*In vivo*	—	Bianchi et al. (2018)
3′,4′,5,7-Tetra-Oquercetin	29.9 mg	—	Stability indicator for hydrolytic degradation	*In vivo*	—	Bianchi et al. (2018)
3′,4′-Di-O quercetin	38 mg	—	Stability indicator for hydrolytic degradation	*In vivo*	—	Bianchi et al. (2018)
Quercetin + maleic anhydride derivatives	50 mM	ROS, caspase-3, -9, and cytoskeletal actin	Cytotoxic effect, Induced apoptosis, Cell cycle arrest, and modification in cytoskeletal actin and nucleus morphology	*In vitro*	HuH7, HepG2	Carrasco-Torres et al. (2017)
Quercetin	25 μg/ml	IGF2BP1, 3, and miR-1275	Reduced viability	*In vitro*	Huh-7	Shaalan et al. (2018)
nano prototype + quercetin	0.10, 20, 50, and 100 mM	IC50s	Induced Apoptosis, necrosis, and antiproliferative effects	*In vitro*	HepG2	Abd-Rabou and Ahmed (2017)
Quercetin-3-O-rutinosidequercetin, -glucoside	2.5–100 μg/ml	—	Cytotoxic effects against cancer cells	*In vitro*	HEPG2	Sobral et al. (2017)
Quercetin	0.67 μM	—	Weak cytotoxic effects against cancer cells and antioxidant effects	*In vitro*	HepG2, Hep3B	Ma et al. (2017)
Quercetin	100 mg/kg	HSP70	Induced apoptosis	*In vivo*		Ma et al. (2017)
Quercetin nanoparticlee	1–50 μM	—	Inhibited tumor growth effect	*In vivo*	HepG2	Wang et al. (2016a)
	*In vitro*					
Quercetin	6.25–100 μM	HDAC8	Cytotoxic effects	*In vitro*	HepG2	Mira and Shimizu (2015)
Quercetin	5–200 µM	GLUT-1 and BAX/BCL-2	Induced apoptosis	*In vitro*	HepG2, HuH7, and Hep3B2.1–7	Brito et al. (2016)
Quercetin	40 mg/kg	Bad, Bax, Bcl-2, and survivin	Induced apoptosis, enhanced 5-FU efficacy, and antiproliferative effects	*In vitro* and *In vivo*	HepG2 and SMCC-7721	Dai et al. (2016)
Quercetin-3-O-glucoside	20–500 μg/ml	—	Antioxidant, cytotoxicity, and induced apoptosis	*In vitro*	HepG2	C. Maiyo et al. (2016)
Quercetin	0–100 µM	PI3K, PKC, ROS, COX-2, p53, and BAX	Cytotoxicity and anticarcinogenic actions	*In vitro*	HepG2	Maurya and Vinayak (2015)
Quercetin	0–50 µM	F-actin	Induced apoptosis and cell cycle arrest	*In vitro*	HepG2	Pi et al. (2016)
Quercetin-3-O-glucoside	100 µM	Caspase-3 and DNA topoisomerase II	Antiproliferative effects, cell cycle arrest, and induced apoptosis	*In vitro*	HepG2	Sudan and Rupasinghe (2015)
Quercetin	0–100 µM	Specificity protein 1 (Sp1)	Induced apoptosis and antiproliferative effects	*In vitro*	HepG2	Lee et al. (2015c)
Quercetin-3-O-glucoside	1–200 μM	Human DNA topoisomerase II and caspase-3	Antiproliferative effects, antioxidant effects, cell cycle arrest, and induced apoptosis	*In vitro*	HepG2	Sudan and Rupasinghe (2014)
Nanocapsulated quercetin	8.98 μmol/kg	TNF-α, IL-6, and MMP-13	Controlled diethylnitrosamine-induced carcinoma	*In vivo*	—	Mandal et al. (2014)
Quercetin	1, 5, 10, 20, and 50 mM	—	Cytotoxicity	*In vitro*	HepG2	Varshosaz et al. (2014)
Quercetin	1–50 mM	—	Anticancer effects	*In vitro*	HepG2	Varshosaz et al. (2014)
Quercetin	1–50 mM	—	Anticancer effects	*In vitro*	HepG2	Varshosaz et al. (2014)
Quercetin	50 μM	P16	Antiproliferative effects and induced apoptosis	*In vitro*	HepG2	Zhao et al. (2014)
Quercetin	1–10 μg/ml	-	Cytotoxicity	*In vitro*	HepG2	Mohamed Al-Taweel et al. (2012)
Quercetin	5 μg/ml	-	Anti-inflammatory and antioxidant	*In vitro*	HepG2	Isa et al. (2012)
Quercetin	50 μmol/L	Heat shock proteins-90, 70, 90α, 76, 60, aand 27	Antiproliferation and inhibited all heat shock proteins	*In vitro*	HepG2	Zhou et al. (2011)
Quercetin	50 μM	Akt, pAkt, Bcl-2, caspase-3, and -9	Induced apoptosis	*In vitro*	HepG2 and Hep3B	Sharma and Bhat (2011)
Quercetin + BB-102	3.125–100 μmol/L	p53, GM-CSF, and B7-1	Antiproliferation and induced apoptosis	*In vitro*	BEL-7402, HuH-7, and HLE	Shi et al. (2003)
Nanoliposomal quercetin	100 mg/kg/d	—	Induced apoptosis and inhibited formation of malignant ascites	*In vivo*	—	Yuan et al. (2006)
Quercetin dissolved in DMSO	0, 40, 60, or and 80 μM	—	Enhanced apoptotis cell cycle arrest	*In vitro*	HA22T/VGH HepG2	Chang et al. (2006)
Quercetin and/or Ni nanoparticles	5.0, 25 and 50 μmol/L	—	Antiproliferative effects	*In vitro*	SMMC-7721	Guo et al. (2009)
Quercetin	0–200 µM	DR5, c-FLIP, and Bcl-xL	Recovered TRAIL sensitivity and induced apoptosis	*In vitro*	HepG2, SK-Hep1, SNU-387, and SNU-449	Kim et al. (2008)
ANBE includes quercetin	100 and 200 mg/kg	CAT, SOD, GPx, GST, ALT, ALP, TBL, AFP, and CEA	Antioxidant effects and induced apoptosis	*In vivo*	—	Singh et al. (2009)
Quercetin	200 mg/kg	p53	Decreased oxidative stress	*In vivo*	—	Seufi et al. (2009)
Quercetin	40 and 80 μM	SOD and MnSOD	Antiproliferative effects and induced apoptosis	*In vitro*	HA22T/VGH HepG2	Chang et al. (2009)
Quercetin	22 µL	p27(Kip1)	Induced apoptosis, cell cycle arrest, and inhibited topoisomerase IIα activity	*In vitro*	HepG2	Naowaratwattana et al. (2010)
Quercetin	0–100 μM	CYP1A1	Increase cytotoxicity, protective effect against DNA strand breaks, and antioxidant activity	*In vitro*	HepG2	Kozics et al. (2011)
Quercetin, nanoencapsulated quercetin	8.98 and 1.898 mmol/ml	Cytochrome c	Antiproliferative effects, antioxidant activity, and induced Apoptosis	*In vivo*	—	Ghosh et al. (2012)
Quercetin	8 *μ*g/ml	PI3K-AKT	Inhibited proliferation	*In vitro*	HepG2 and Huh-7	Pan and Pan (2021)
Quercetin	50 mg/kg	P16	Ineffective against age-associated NAFLD-induced HCC	*In vitro* and *In vivo*	DEN/HFD mouse model	Raffaele et al. (2021)
Quercetin	100 μg/ml	PEPCK and G6Pase	Antioxidant effect	*In vitro*	HepG2	Pasachan et al. (2021b)
Quercetin	100 mg/kg	Nrf2/Keap1 pathway	Antioxidant effect	*In vitro* and *in vivo*	HepG2 and male Kunming mice	Zhang et al. (2020)
Quercetin	100 μM	—	Antiproliferative effect, induced apoptosis, G_0_/G_1_, G_2_/M, and S phase cell cycle arrest	*In vitro*	KIM-1, HAK-1A, HAK-1B, HAK-2, and HAK-3	Hisaka et al. (2020)
Quercetin	3, 7 μM	TGF-α, p38 MAPK, and AKT	Suppressed migration	*In vitro*	HuH7	Yamada et al. (2020)

### 3.6 Quercetin and Oral Cancer

Singh et al. (2020) investigated the effects of a combination of quercetin and resveratrol (at minimum non-toxic concentrations) on oral cancer. In their study, the combination caused cell death in Cal-33 oral cancer cells but not in HEK-293 normal cells. They found that stimulation of cyclin E (a cell cycle regulatory protein) and reduced expression of cyclin A led to cell cycle arrest at the S phase in Cal-33 cells. The comet assay in association with gamma-H2AX foci showed DNA injury, and the overexpression of Bax and PARP1 cleavage showed cellular apoptosis after the combination treatment. Resveratrol treatment, both alone and in combination, could downregulate the expression of histone deacetylase 1 (HDAC), HDAC8, and HDAC3. Collectively, the combined quercetin and resveratrol regimen suppressed cell growth and caused DNA damage and cell cycle arrest in oral cancer cells but not in non-cancer cells (Singh et al., 2020).

Yuan and others designed a study to identify the effects of quercetin on proliferation, migration, apoptosis, and invasion in the vincristine (VCR)-resistant P glycoprotein (P-gp) overexpressing oral cancer cell line, KB/VCR. The VCR sensitivity of KB/VCR cells was increased by inhibiting P-gp expression with quercetin. They found that the proliferation, invasion, and migration of KB/VCR cells were suppressed by quercetin, in addition to enhanced VCR sensitivity and apoptosis (Yuan et al., 2015). The anticancer activity of these agents was assessed in KB/VCR oral cancer cells *in vitro*. According to their study, 25–100 µM concentrations of quercetin could efficiently block proliferation, invasion, and migration of KB/VCR cells, while at concentration above 50 µM, it resulted in the G1 phase cell cycle arrest. According to apoptosis analysis, 50 or 100 µM of quercetin stimulated apoptosis in KB/VCR cells by inhibiting Bax expression and upregulating Bcl-2 and caspase-3. Dose ranges of 25–100 µM quercetin reduced drug efflux mediated by decreasing the expression of P-gp in KB/VCR cells. A combination of quercetin (50 µM) and vincristin (0.375 µM) was even more effective in inducing apoptosis. Consequently, quercetin may reduce drug resistance as a promising approach for treatment of oral cancer and other tumor types (Yuan et al., 2015).

In another study by Ma et al., the effects of quercetin on the human oral cancer SAS cell line were evaluated. They found that quercetin stimulated apoptosis by mitochondrial and endoplasmic reticulum stress signaling pathways in SAS cells (Ma et al., 2018). The effect of quercetin on cell death was investigated using flow cytometry, Western blotting, confocal laser microscopy, and Annexin V/propidium iodide (PI) double staining, at 6–48 h following quercetin treatment. They found that quercetin reduced cell viability and increased production of Ca2+ and reactive oxygen species. Annexin V/PI staining showed increased apoptosis, with a reduction in mitochondrial membrane potential (ΔΨm), in addition to modified expression of apoptosis-related proteins. Western blotting showed that quercetin increased Fas, Fas ligand, Fas-related protein with death domain, and caspase-8, which were correlated with death receptors on the surface of the cells. Quercetin upregulated ATF-6α (activating transcription factor), gastrin-releasing peptide-78, and ATF-6β, showing endoplasmic reticulum stress. The levels of pro-apoptotic protein BH3-interacting death-domain antagonist in conjunction with reduced expression of antiapoptotic proteins Bcl-2 and Bcl-extra-large protein, all of which reduced mitochondrial membrane potential. In addition, confocal microscopy showed that quercetin upregulated the expression of apoptosis-inducing factor, cytochrome c, and endonuclease G, all of which can stimulate apoptosis. Therefore, quercetin may be a promising anticancer drug in the treatment of oral cancer (Ma et al., 2018).

Huang et al. (2013) explored the antitumor effects of quercetin in human oral cancer cell lines with EGFR upregulation and assessed the role of FOXO1 transcription factor in growth inhibition induced by quercetin. They showed that FOXO1 played a critical role in suppressing cellular growth mediated by quercetin in oral cancer cells with high EGFR expression. Quercetin results caused cell cycle arrest at the G2 phase and stimulated apoptosis in TW206 and HSC-3 oral tumor cells with high EGFR expression. Quercetin activated FOXO1 and blocked EGFR/Akt activation. Silencing of FOXO1 decreased the expression of FasL and p21 mediated by quercetin and further increased apoptosis and G2 cell cycle arrest. Similarly, quercetin inhibited cancer growth in the mouse HSC-3 xenograft model (Huang et al., 2013).

Cheng et al. (2012) focused on the effects of quercetin on overcoming drug resistance and the underlying mechanisms in oral squamous cell carcinoma. They used a non-adhesive cell culture system, called the DRSP (drug-resistant sphere) model, in order to generate drug-resistant cells from oral tumor cell line SCC25. A comparative analysis was conducted between DSRPs and the parental control cells based on the measurement of EMT (epithelial-mesenchymal transition)-related markers, CSC features, and genes related to drug resistance *in vitro*. They also looked at carcinogenesis and tumor treatment *in vivo*. Their study on DRSPs showed overexpression of genes involved in drug resistance, including CSC-related markers and ABCG2, such that DRSPs had higher cisplatin (Cis) resistance and more CSC features than those in the control group. Furthermore, upregulation of p-Hsp27 (phosphorylated heat-shock protein 27) due to activation of the p38 MAPK signaling pathway was observed in DRSPs. Hsp27 silencing reduced the resistance to cisplatin and triggered apoptosis in DRSPs. Moreover, quercetin reduced the expression of p-Hsp27 and acted as an Hsp27 inhibitor to reduce the EMT status, resulting in the activation of apoptosis in DRSPs. Enhanced tumorigenesis was verified in DRSPs using a nude mouse xenograft model. Combined treatment by cisplatin and quercetin could reduce tumor growth and decrease drug resistance in OSCC. The p38 MAPK–Hsp27 signaling axis plays a major function in drug resistance induced by CSCs in oral cancer, and this could be inhibited by quercetin and cisplatin treatment (Cheng et al., 2012). Table 6 lists some studies on the therapeutic effects of quercetin in oral cancer.

**TABLE 6:** Studies on the therapeutic effects of quercetin in oral cancer.

Type of Quercetin	Dose	Targets	Results	Model (*in vitro/in vivo*/Human)	Cell Line	Reference
Quercetin	25–400 μM	NF-κB and matrix metalloproteinase-2/-9	Inhibited migration and invasion	*In vitro*	SAS	Lai et al. (2013)
Quercetin	10, 50, and 200 µM	SGLT1 and MRP2	Induced apoptosis	*In vitro*	SCC-9	Browning et al. (2005)
Quercetin	10–100 µM	Blc2, Bax, and caspase 3	G1 phase cell cycle arrest and induced mitochondrial apoptosis	*In vitro*	SCC25	Chen et al. (2013)
Quercetin	5–200 µM	Caspase-3, S-Phase, and TS enzyme	Induced necrosis and apoptosis	*In vitro*	SCC-9	Haghiac and Walle (2005)
Quercetin	0–50 µM	FOXO1 and EGFR	Growth arrest and apaptosis	*In vitro* and *in vivo*	HSC-3, TW206, and HGF	Huang et al. (2013)
Quercetin	20, 40, and 80 µM	Anti-PARP antibody and caspase-3	Induced apoptosis	*In vitro*	SCC-1483, SCC-25, and SCC-QLL1	Kang et al. (2010)
Quercetin	40 µM	ROS, caspase 3, 8, 9, Fas, Fas ligand, and ATF-6β	Induced morphological changes, decreased viability, and induced apoptosis	*In vitro*	SAS	Ma et al. (2018)
Quercetin	100 mM	p38 MAPK–Hsp27, ABCG2, and MDR1	Reduced tumor growth and decreased drug resistance	*In vitro* and *in vivo*	SCC25	Chen et al. (2012)
Quercetin	1–25 µM	cytochrome P450 and 1B1	Chemopreventive agent	*In vitro*	SCC-9	Walle and Walle (2007)
Quercetin	25–100 µM	Bcl-2, Bax, and caspase-3	Inhibited proliferation, inhibited migration and invasion, and induced apoptosis	*In vitro*	KB/VCR	Yuan et al. (2015)
Quercetin	0, 50, and 100 µM	miR16 and HOXA10-axis	Inhibited viability and migration and invasion	*In vitro*	HSC-6, SCC-9, and hNOK	Zhao et al. (2019)
Quercetin	10 μM	Cyclin A, E, PARP1, and Bax	Inhibited proliferation, cell cycle arrest, and DNA damage	*In vitro*	SCC-15 and Cal-33	Singh et al. (2020)

## 4 Delivery of Quercetin

Quercetin and flavonoids have been used in the treatment of several human diseases, and they may be found in a wide range of foods and dietary supplements. Quercetin and its related compounds can have a wide range of beneficial effects, and numerous studies have been designed to overcome the drawbacks, such as low oral absorption and poor water solubility. Quercetin has low skin permeability, and some drug formulations have been produced to increase its permeability for topical application, such as nanocapsules, nanoemulsion, microemulsions, and solid–lipid nanoparticles (Nagula and Wairkar 2019).

Different delivery systems for flavonoids have been designed based on nanoformulations, which may enhance the efficacy of these molecules (Hariharan et al., 2006; Vicentini et al., 2008; Yen et al., 2009). For instance, nanoparticles, microemulsions, solid lipid nanoparticles, and liposomes have been widely used for different applications, particularly for neurodegenerative diseases and aging (Priprem et al., 2008; Wu et al., 2008).

When hydrophobic drugs are encapsulated within nanoparticles, they become completely dispersed in an aqueous environment and can then be administered intravenously. Antitumor drugs can be delivered to the tumor microenvironment when they are incorporated inside biodegradable polymeric nanoparticles, which have been explored as a novel drug delivery system in clinical trials (Park et al., 2009). For instance, PCL/PEG (poly-3-caprolactone)-polyethylene glycol) is a readily obtained biodegradable agent with amphiphilic properties that can be used as a drug delivery system (Gou et al., 2011). The use of PCL/PEG nanoparticles to increase the water solubility of hydrophobic compounds has been widely explored in recent studies. In a study by Gao et al. (2012) the effects of a quercetin nanoformulation on colon carcinogenesis and the proliferation of ovarian cells were investigated. Quercetin was encapsulated into biodegradable micelles made of monomethoxy-poly (ethylene glycol)-poly (ε-caprolactone) (MPEG-PCL) for the treatment of ovarian cancer. These MPEG-PCL micelles were loaded with quercetin (QU/MPEG-PCL) with a drug loading capacity of 6.9% and an average particle size of 36 nm and allowed the complete dispersion of quercetin in water.

Lipid nanocapsules (LNCs) can also be used for quercetin encapsulation because their lipid-content will dissolve quercetin. In a study by Hatahet et al., they prepared spherical lipid nanocapsules containing Cremophor EL ranging in size from 26 ± 3 nm to 54 ± 3 nm (Hatahet et al., 2017). The introduction of Cremophor El allowed better encapsulation of quercetin because this compound contains OH groups that interact with quercetin polar groups. The water solubility of quercetin increased up to 5,000 times, with adequate thermal stability at 4 and 25°C. Less than 15% of the quercetin loaded into LNC was released after 1 day, which suggests a long-term controlled delivery into the skin. Quercetin LNC’s anti-inflammatory activity was assessed in THP-1 cells (leukemic monocytes), which showed that there was no cytotoxicity, and quercetin may exert its anti-inflammatory effects by removing free radicals from the skin, thus protecting epidermal keratinocytes. Moreover, LNCs loaded with quercetin reduced reactive oxygen species and decreased inflammation (Hatahet et al., 2017).

MOFs (metal organic frameworks) have been studied recently as drug delivery vehicles as they possess a regular pore structure, significant pore volume, adequate chemical and thermal stability, and easy surface functionalization (Horcajada et al., 2008; Sun et al., 2011; Chen et al., 2014). Among these compounds, ZIF-8 (zeolitic imidazolate framework-8) is a biocompatible metal organic framework made up of 2-methylimidazolate and zinc ions. ZIF-8 has suitable stability in various physiological conditions but will disintegrate in the tumor tissue due to the lower extracellular pH. Therefore, ZIF-8 may serve as a novel drug delivery vehicle (Venna et al., 2010; Sun et al., 2012; Zhang et al., 2017b). PTT (photothermal therapy) employs light-induced hyperthermia to destroy the target tumor cells, as a new approach in cancer therapy (Liang et al., 2016; Li et al., 2017; Sheng et al., 2018). Much effort has been made to synthesize nanoparticles (NPs) as chemo-PTT platforms to improve the therapeutic efficacy and to overcome drug resistance in tumor cells (Yao et al., 2017a; Yao et al., 2017b; Tian et al., 2017). For instance, CuS NPs have been used as PTT agents and chemo-PTT nanocomposites (Gao et al., 2016; Liu et al., 2017b; Ren et al., 2017). These CuS NPs could be encapsulated in the ZIF-8 framework without changing the drug loading capacity or altering the pH sensitivity (Wang et al., 2016b). Jiang et al. (2018) asked whether quercetin could be loaded into ZIF-8, considering the opposite charges and the coordination effect between ZIF-8 and quercetin. In their study, a combined platform with quercetin as an antitumor agent and CuS NPs as a PTT agent was synthesized based on folic acid–bovine serum albumin (FA–BSA)-modified ZIF-8. Their study showed that the low water solubility, poor bioavailability, and chemical instability of quercetin could be improved by using FA–BSA/CuS@ZIF-8 as a drug delivery vehicle. According to *in vivo* NIRF imaging results, the tumor uptake was significantly increased by using the FA–BSA-modified MOF. Moreover, FA–BSA/CuS@ZIF-8-QT had minimal hemolysis and only minor toxic effects to normal organs, suggesting it could be safe for intravenous administration. Furthermore, the *in vivo* studies showed that the FA–BSA/CuS@ZIF-8-QT combined with laser irradiation showed a higher anticancer effect than PTT or chemotherapy alone, leading to significant inhibition of tumor growth. They concluded that FA–BSA/CuS@ZIF-8-QT could be a novel multimodal nano-platform for the administration of quercetin in cancer therapy (Jiang et al., 2018).

## 5 Conclusion

There are a wide range of treatment options for GI tumors. These treatment strategies may be used alone or combined with adjuvant or other therapies. Up to now, the available treatment strategies for GI tumors include: (Miller et al., 2020) surgical resection, the removal of the solid mass in patients without advanced disease; (Schiller and Lowy 2021) chemotherapy, the administration of cytotoxic drugs to destroy malignant cells; (Patel 2020) radiotherapy, used in the treatment of localized solid lesions; and (Colli et al., 2017) hormonal therapy, a systemic therapeutic approach targeting tumor cells all over the body. Nevertheless, effective tumor treatment often necessitates highly coordinated combinations of different therapeutic strategies. This approach is known as multi-modality treatment, including surgical resection, chemotherapy, radiotherapy, and hormone therapy. Since quercetin is able to exert major anticancer effects on GI tumors, this agent can be considered to be a promising antitumor natural compound. However, some questions regarding its application require further study. Some of its limitations comprise inactive metabolic products, poor aqueous solubility, insufficient intrinsic activity, poor absorption, and excessive metabolism. On the other hand, metabolic conversion of quercetin can significantly alter its efficacy and bioavailability. Similar to other xenobiotics, the reactivity of quercetin may be attenuated by conjugation. A number of quercetin-conjugated metabolites have a biological function. Aglycone-conjugated quercetin metabolites are synthesized by the site-specific action of certain enzymes, and several biological targets have been identified. As a result, more detailed studies need to be performed to improve our understanding of quercetin and its active metabolites, and their underlying mechanism of action. Moreover, nanostructured carriers including, liposomes, polymers, chitosan, and other nanoparticles may be effectively used to deliver quercetin and active metabolites to various tissues. These nanocarriers can increase the solubility of polyphenols in water, prolong the circulation time, improve the absorption rate, and the target-specificity. However, although quercetin delivery *via* these carriers has not been much investigated, it may be a useful approach to improve the activity of polyphenols in different types of cancer, especially in colorectal cancer. Additionally, further investigations are needed to identify any synergistic effects of quercetin when combined with other antitumor drugs. Moreover, clinical trials investigating the preventive and therapeutic activity of quercetin in GI cancers have high priority. More data for future epidemiological studies may be obtained by updating of the dietary flavonoids databases. Finally, although quercetin has shown some promising results in the prevention and treatment GI tumors, further studies are still needed to thoroughly understand the underlying mechanisms and any possible adverse effects.
